# Dietary Energy Levels Affect Growth Performance through Growth Hormone and Insulin-Like Growth Factor 1 in Yak (*Bos grunniens*)

**DOI:** 10.3390/ani9020039

**Published:** 2019-01-28

**Authors:** Chao Yang, Jianbo Zhang, Anum Ali Ahmad, Pengjia Bao, Xian Guo, Ruijun Long, Xuezhi Ding, Ping Yan

**Affiliations:** 1 Key Laboratory of Yak Breeding Engineering, Lanzhou Institute of Husbandry and Pharmaceutical Sciences, Chinese Academy of Agricultural Sciences, Lanzhou 730050, China; yangchaocas@126.com (C.Y.); zhangjb9122@163.com (J.Z.); baopengjia@caas.cn (P.B.); guoxian@caas.cn (X.G.); 2 State Key Laboratory of Pastoral Agricultural Ecosystem, College of Pastoral Agriculture Science and Technology, Lanzhou University, Lanzhou 730020, China; 3 School of Life Sciences, Lanzhou University, Lanzhou 730000, China; anum2017@lzu.edu.cn (A.A.A.); longrj@lzu.edu.cn (R.L.)

**Keywords:** Yak, dietary energy level, growth performance, growth hormone (GH), insulin-like growth factor 1 (IGF-1)

## Abstract

**Simple Summary:**

Yak is always in a malnutrition condition under pure grazing in the cold season. Many studies have suggested that provision of supplementary feed to yak cannot only be an effective approach to avoid weight loss, but also improve productivity. Growth hormone (GH) and insulin-like growth factor 1 (IGF-1) could improve yak productivity. In current work, we evaluated the effects of different energy feedstuffs on the serum concentration of GH, IGF-1, and relative hepatic expression of their associated binding proteins and receptors, as well as growth performance of yak. The results indicated that average daily gain (ADG), body weight (BW), feed conversion ratio, serum IGF-1 concentration, and relative expression of IGF-1 and IGFBP-3 were increased with an increase in dietary energy level, while serum GH concentration and hepatic growth hormone (GHR) expression were decreased. Serum IGF-1 concentration, relative hepatic expression of IGF-1, and insulin-like growth factor binding protein 3 (IGFBP-3) showed positive correlation with ADG, but serum GH concentration and GHR expression were negatively correlated with ADG. Yak offered with medium energy diet (NEg: 6.2 MJ/kg) displayed better growth by gaining 0.883 kg/d and showing a superior feed conversion ratio during experiment.

**Abstract:**

The objective of this study was to investigate the effects of different dietary energy levels on serum concentrations of growth hormone (GH) and insulin-like growth factor 1 (IGF-1), as well as gene expression of their associated binding proteins and receptors in yak. Fifteen adult male yaks with BW of 276.1 ± 3.5 kg were allotted in three dietary groups and were fed with low (LE), medium (ME), and high energy (HE) level diet having different NEg of 5.5 MJ/kg, 6.2 MJ/kg, 6.9 MJ/kg, respectively. The effects of these treatments on ADG, BW, ADFI, and feed conversion ratio were significant (*p* < 0.05) throughout the experimental period. Serum GH concentration decreased (*p* < 0.05) with an increase in dietary energy level on d 30 and d 60. While IGF-1 concentration was higher (*p* < 0.05) in ME group, as compared to LE and HE groups on d 60. The expression level of growth hormone receptor (GHR) was decreased (*p* < 0.001) and IGF-1 was increased with the increase in the dietary energy level. The relative expression of insulin-like growth factor binding protein 3 (IGFBP-3) was higher (*p* < 0.001) in ME and HE groups, except the LE group. In conclusion, our findings provide a first insight into the combined effect of GH and IGF-1 in controlling the metabolism and productivity of yak. It also showed that medium energy level diet contributed to promote growth performance of yak during the cold season.

## 1. Introduction

Yak (*Bos grunniens*) is a multi-tasker grazing livestock species offering meat, milk, hides, transportation, and dung as fuel for the Tibetan nomads exclusively in the Hindu Kush-Himalayan region and the Qinghai-Tibetan Plateau [1]. Due to short plant growing season (90–120 days), the adult yaks under traditional grazing system always lose 25–30% of their live-weight during long cold seasons [2]. Energy is essential for animals to support their normal physiological activities such as walking, grazing, rumination, digestion, and heat production, which primarily comes from dietary carbohydrates and secondary from the mobilization and catabolism of body reserves. Under conditions of low ambient temperature, animals always increase their heat production by using feed energy or fat mobilization to continue normal physiological activities, which in turn decreases their growth performance and production efficiency. High-energy diets have been reported to promote growth performance of goats [3], sheep [4], cow [5], and beef cattle [6]. Beauchemin et al. [7] demonstrated that decrease in the dietary energy level reduced the growth rate and growth efficiency in lambs. Additionally, our recent research documented that increase in dietary energy level enhanced fat deposition and synthesis of fatty acid via upregulating the expression of lipogenic genes in yak [8].

A previous study demonstrated that increase in dietary energy level could significantly improve feed conversion ratio, serum concentration of growth hormone (GH), and insulin-like growth factor 1 (IGF-1) in cattle by reducing feed intake [9]. GH and IGF-1 have an independent, as well as combined, impact on controlling metabolism and production of animals. In details, GH is synthesized in pituitary gland and acts directly on liver and adipose tissue to regulate gluconeogenesis, proteosynthesis, lipogenesis, lipolysis, and insulin secretion by binding to growth hormone receptor (GHR) [10,11,12]. IGF-1 is a critical somatomedin synthesized in liver and regulates the function of each organ. It plays an important role in some physiological processes, contributes to improve feed conversion rate, and also increases protein synthesis [13]. IGF-1 binds to insulin-like growth factor binding protein-3 (IGFBP-3) to influence the growth, development, and reproduction in animals [14]. Meanwhile, the existence of axis between GH and IGF-1 has played a vital role in the regulation of metabolism and GHR combines with GH to stimulate a series of metabolic activities by producing IGF-1 in the target tissues, especially in liver [15,16]. Many researchers have reported that yak has developed special strategy to adapt to the shortage of nutrition and hypoxia stress on gene and morphology levels [17,18,19]. However, unfortunately there is less information available about the effects of different dietary energy levels on growth performance and GH and IGF-1 regulated growth performance of yak. Therefore, the aim of this study was to investigate the effect of different dietary energy levels on growth performance of yak by measuring GH and IGF-1 contents.

## 2. Materials and Methods

The use of animals was approved by Gansu Province Animal Care Committee (Lanzhou, China), and experimental procedures in this study were in accordance with the Guide for the Care and Use of Laboratory Animals. The feeding trials were conducted between November 2015 and January 2016 at Hezuo (35°09′ N, 102°99′ E) of Gannan Tibetan Autonomous Prefecture, Gansu Province, China. Hezuo is located in the South-Eastern of Qinghai-Tibetan Plateau with the average altitude around 2936 m, a representative plateau-climate and an annual average temperature around 1.7 °C. The average temperature and relative humidity in the barn during whole experimental period were 3 °C and 42%, respectively.

### 2.1. Animals and Experimental Diets

Fifteen adult 4-year-old male yaks with initial average weight of 276.1 ± 3.5 kg were divided into five blocks, within each block three yaks with similar initial body weight (BW) were assigned randomly to three dietary treatments. All yaks were individually housed in tethered stalls in a good ventilated barn, with 6 m^2^ per animal and floor was paved with dry wheat straw, which was replaced three times a week. Two feed troughs were fixed with beton for each animal. The barn was thoroughly sanitized before animals were housed in and was cleaned every morning. Diets were formulated by following the recommendations of Feeding Standard of Beef Cattle (2004, [20]) with recommendatory NEg of 10.76 MJ/d for fattening cattle (BW 300 kg and ADG 1.0 kg/d). The diets were isonitrogenous, had forage-to-concentrate ratio of 70:30 including same roughage (highland barley hay) as basic diet and three different concentrates (on a DM basis): Low energy diet (LE: 5.5 MJ/kg), medium energy diet (ME: 6.2 MJ/kg), and high energy diet (HE: 6.9 MJ/kg). The nutrition compositions of three energy diets are shown in Table 1.

### 2.2. Experimental Procedure and Analytical Methods

The animals were adapted to each treatment for two weeks prior to a 60-day experimental period. The animals were fed ad libitum in two equal portions at 08:00–09:00 am and 17:00–18:00 pm on daily basis with 2.5 kg concentrates and 6.0 kg roughage. All the animals had free access to water. The amount of diet received by each animal was recorded to calculate average daily feed intake (ADFI), and ADFI was determined as the total feed intake/60. Feed troughs were cleaned every morning before feeding the animals, orts samples were collected once a week, weighted and stored at −20 °C. The samples were dried in the lab oven at 65 °C for 48 h and the oven-dried samples were grounded to pass 2-mm screen for analysis of DM (105 °C for 8 h), crude protein, crude fat, calcium and phosphorus with relevant AOAC methods [21]. Neutral detergent fiber (NDF) and acid detergent fiber (ADF) were measured using the method of Van Soest et al. [22]. 

Yaks were weighed on day 1, day 30, and at the end of the experiment in the morning before feeding and the records were used to determine average daily gain (ADG), while ADG was calculated as (day 60 weight, day 1 weight)/60. The feed conversion ratio was calculated as total feed intake/total weight gain. All animals were transferred to slaughter house and humanely slaughtered by exsanguination after electrical stunning at the end of the trial.

### 2.3. Sample Collection and Analysis

Blood samples (15 mL) were collected via jugular venipuncture in ethylenediaminetetraacetic acid dipotassium salt-containing tubes (Jiangsu Yuli Medical Instrument Co, Taizhou, China) of each animal without feeding between 08:00 am and 10:00 am on d 30 and d 60, then centrifuged at 3000 rpm for 15 min at 4 °C. Serum samples were stored at −20 °C for subsequent IGF-1 and GH concentrations analysis by using commercial ELISA kit (Beijing Sino-uk institute of Biological Technology, Beijing, China). Liver samples were immediately collected in quadruplicate after slaughtering, washed with 0.9% NaCl solution, stored in 2 mL cryogenic vials (Corning Incorporated, New York, USA) and snap frozen in liquid nitrogen. All liver tissues were stored at −80 °C in laboratory until total RNA extraction.

### 2.4. RNA Extraction and Quantitative Real-time PCR

The gene names, sequences, accession numbers, primer sequences for *Bos grunniens* GHR, IGF-1, and IGFBP-3 are listed in Table 2.

Total RNA from liver tissues was extracted by using RNAiso Plus kit (TaKaRa, Dalian, China) according to the manufacturer’s instructions. RNase free pipet tips, 1.5 mL microtubes and PCR tubes (Axygen, MA, USA) were used. The extracted RNA was dissolved in 40 μL diethylpyrocarbonate (DEPC)-treated water (Solarbio LIFE SCIENCES, Beijing, China) and concentration was measured by spectrophotometer (NanoDrop 2000, Thermo Scientific, MA, USA) at 260/280 nm (OD260:OD280 = 1.8~2.0). To check purity and integrity of RNA, agarose gel electrophoresis having 1 pg/mL ethidium bromide was used to stain 28S and 18S bands. Total RNA was reversely transcribed in three replicates using a PrimeScript™ RT reagent kit (TaKaRa, Dalian, China) in 20-μL reaction volume. In brief, possible gDNA was removed through 2.0 μL 5×gDNA Eraser Buffer, 1.0 μL gDNA Eraser, 2 μL total RNA (200 ng/μL) and 5 μL RNase Free dH_2_O at room temperature for 5 min. Then reverse transcription reaction was performed on a thermal cycler (DNA Engine Dyad Peltier Thermal Cycler, Bio-Rad, CA, USA) with 10 μL gDNA purified sample, 1.0 μL PrimeScript RT Enzyme Mix I, 1 μL RT Primer Mix, 4 μL 5×PrimeScript Buffer 2 (for Real Time) and 4 μL RNase Free dH_2_O by using following program: 37 °C for 15 min, 85 °C for 5 s and 4 °C for an indefinite time. RT-PCR products were stored at −20 °C until quantitative real-time PCR was performed.

Quantitative real-time PCR was performed in triplicate to determine relative mRNA expression using SYBR® Premix Ex Taq™ II (TaRaKa, Dalian, China). Each 20-μL real-time reaction contained 10 μL SYBR Premix Ex Taq II (2×), 0.8 μL each of 10 μM primers, 1.0 μL cDNA and 7.4 μL RNase Free dH_2_O. Reactions were run on a fluorescence thermal cycler (CFX96, Bio-Rad, CA, USA) and following program was used: 95 °C for 30 s, 40 cycles of 95 °C for 5 s, with respective annealing temperature (Table 2) for 30 s and a melting curve of increasing temperature of 0.5 °C every 5 s starting from 65 °C. The quantitative RT-PCR analysis for studied genes was performed in triplicates using cDNA from five animal replicates in group. The threshold cycle (CT) resulting from quantitative RT-PCR was analyzed using the 2^−ΔΔCt^ method, and all the data were normalized with beta-actin (β-actin) used as a reference gene. 

### 2.5. Statistical Analysis

Data for BW and ADG were analyzed using the repeated measures procedure with the time taken as repeated measures by PROC MIXED model in SAS 9.4 (SAS Inst. Inc., Cary, NC, USA). The model used for the analysis was as follows:

Y = μ + T_i_ + D_j_ + B_l_+ TD_ij_ + Y_ik_ + ε_ijk_,

in which Y is the dependent variable, μ is the population mean for the variable, T_i_ is the dietary treatments (i = 1, 2, 3) as the fixed effect, D_j_ is experimental day (j = 0, 30, 60) as the fixed effect, B_l_ is the blocks (i = 1, 2, 3…5) as the fixed effect, TD_ij_ is the interaction between treatments and experimental day, Y_ik_ is yak (k = 1, 2, 3…15) as the random effect and ε_ijk_ is the random error associated with the observation of ijkl. The interaction between treatments and experimental day was found for BW and ADG, and then LSD procedure of PROC GLM model was used to conduct multiple comparisons of three treatments on same time or same experimental period using SAS 9.4. The model used for the analysis was as follows:

Y = μ + T_i_ + B_j_ + ε_ij_,

in which Y is the dependent variable, μ is the population mean for the variable, T_i_ is the dietary treatments (i = 1, 2, 3) as the fixed effect, B_j_ is the blocks (j = 1, 2, 3…5) as the fixed effect, ε_ij_ is the random error associated with the observation of ij.

ADFI, feed conversion ratio, serum concentrations of GH, and IGF-1 on day 30 and day 60, and gene relative expressions among three treatments were processed using one-way ANOVA analysis with LSD procedure for multiple comparisons of three treatments using SAS 9.4. Data were described as mean ± SEM (Standard Error of Mean). Significance of effect was declared at *p* ≤ 0.05 and trends at 0.05 < *p* ≤ 0.10. 

The Pearson correlation coefficient was also calculated by using the PROC CORR from SAS 9.4. The correlation analysis was based on individual values and was independent of the treatments. Fifteen observations for each group or index of the study were used for correlation analysis. *, ** and *** indicated *p* < 0.05, *p* < 0.01 and *p* < 0.001, respectively.

## 3. Results

### 3.1. Effects of Dietary Energy Levels on Growth Performance of Yak

BW, ADG, ADFI, and feed conversion ratio on day 1, day 30, day 60, and from day 1~60 of Gannan yaks are presented in Table 3. The effects of dietary treatments (*p* = 0.002), time (*p* < 0.001) and interaction between treatments (*p* = 0.009) on BW were significant BW on day 30 (*p* = 0.037) and day 60 (*p* = 0.012) were significantly increased with increasing dietary energy level. ADG was significantly affected by increasing dietary energy level during three treatments from day 1 to day 60, day 1 to day 30, and day 30 to day 60 (*p* < 0.001), and changing dietary NEg from 5.5 (LE) to 6.9 MJ/kg (HE) significantly increased ADG from 0.833 to 1.186 kg/d in yaks during day 1 to day 30 (*p* < 0.001). A slight decrease in ADG was observed in medium to high dietary energy groups (0.883 vs. 0.860 and 0.660 vs. 0.533, *p* > 0.05), however ME group had a higher ADG than LE group from day 0 to day 60 and day 30 to day 60 (0.624 vs. 0.883 and 0.413 vs. 0.660, *p* < 0.05). Additionally, the effects of experimental time on ADG in this study were noticeable showing lower value on d 30~60 than d 1~30 (*p* < 0.001). The interaction between dietary treatments and time on ADG was significant (*p* = 0.016). ADFI decreased (*p* = 0.01) when a higher energy diet was offered, but LE group displayed higher (*p* = 0.003) ADFI value than HE group. Feed conversion ratio was significantly affected (*p* < 0.001) by increasing dietary energy level and it was higher in LE group than that in ME and HE groups (*p* < 0.05).

### 3.2. Effects of Dietary Energy Levels on Serum GH and IGF-1 Concentrations in Yak

**Figure 1:**
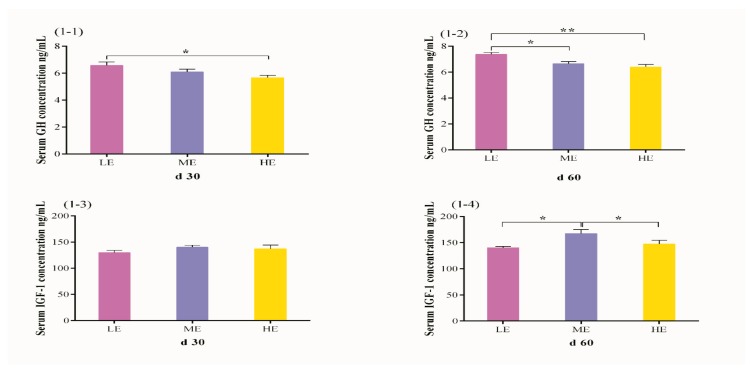
Effects of different dietary energy levels on serum concentration of GH (**1-1**,**1-2**) and IGF-1 (**1-3**,**1-4**) in yak. The sampling day for each group are day 30 and day 60 during the whole experimental period. GH, growth hormone; IGF-1, insulin-like growth factor 1. Values are presented as mean ± SEM (standard error of mean). Within the same day of GH and IGF-1, * and ** indicate *p* < 0.05 and *p* < 0.01, respectively.

The effect of different dietary energy levels on serum GH and IGF-1 concentrations in yak are presented in Figure 1. An increase in dietary energy level decreased the GH concentration under barn feeding conditions (*p* < 0.05). The concentration of GH in LE dietary group was higher (*p* = 0.011) than HE dietary group on day 30 (Figure 1(1-1)) while HE dietary group on day 60 displayed significantly higher concentration (*p* = 0.012 and *p* = 0.002) of GH as compared to ME (*p* = 0.012) and LE (*p* = 0.002) dietary groups. There was no significant effect of dietary energy level on IGF-1 concentration (*p* > 0.05) on day 30 (Figure 1(1-3)), however higher IGF-1 concentration (*p* = 0.010 and *p* = 0.044) was observed in the ME group but no significant difference (*p* > 0.05) was observed between LE and HE dietary groups on day 60 (Figure 1(1-4)).

### 3.3. Effects of Dietary Energy Levels on Hepatic mRNA Abundance of GHR, IGF-1 and IGFBP-3 in Yaks

**Figure 2:**
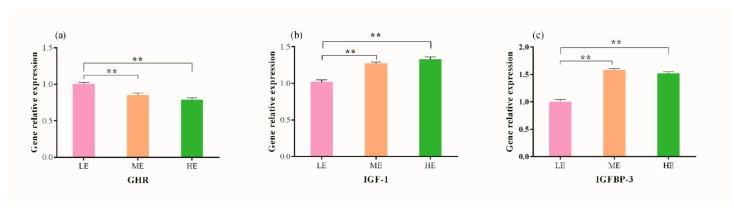
Effect of different dietary energy levels on relative expression of hepatic genes. (**a**) GHR, growth hormone receptor; (**b**) IGF-1, insulin-like growth factors 1; (**c**) IGFBP-3, insulin-like growth factor binding protein 3. Data are shown as mean ± SEM (standard error of mean). Within the same gene, * and ** indicate *p* < 0.05 and *p* < 0.01, respectively.

The relative hepatic expression of GHR significantly reduced (*p* < 0.001) with an increase in dietary energy levels, and yak fed with low energy diet showed high (*p* < 0.001) GHR expression than those offered with medium and high energy diets (Figure 2a). A linear increase in relative expression of IGF-1 was recorded with an increase in dietary energy level. IGF-1 expression was similar (*p* > 0.05) in both ME and HE dietary treatments, however significantly higher values (*p* < 0.001) were observed in both groups as compared to LE dietary group. The mRNA abundance of IGFBP-3 was greater in ME and HE dietary groups than LE group (*p* < 0.001), while no difference was observed between ME and HE dietary groups (*p* > 0.05).

### 3.4. Correlation Analysis between Serum Hormone Levels and Expression Levels of Genes on Growth Performance of Yak

An inverse correlation between GH concentration and ADG was recorded on day 60 (*p* = 0.044), as shown in Table 4. While the concentration of IGF-1 had an opposite tendency with ADG on day 30 (*p* = 0.017) and day 60 (*p* = 0.006). The relationship between relative genes expression and growth performance of yak are shown in Table 5. GHR expression level was negatively correlated (*p* = 0.042) with ADG, while the expression level of IGFBP-3 had significantly positive (*p* = 0.003) correlation with ADG.

## 4. Discussion

The yak evolved as a remarkable domesticated animal having compensatory growth potential to recover its body condition when enough nutrition was available [23] and our previous results demonstrated that extremely well adapted yaks lost substantial BW during cold season. The expenditure of energy during grazing and survival in the harsh environmental conditions, is clear from better BW gain of the yak feed indoors [8]. More recently supplementary feeding of roughages and concentrates is developed as a promising strategy to improve the growth performance of grazing yaks and to prevent them from losing body weight in the legume-deficient alpine region of Qinghai–Tibetan Plateau over the long cold season [1]. The feedstuffs evaluated in this study as the mainstay of Tibetan feedlot industry during the cold season included oat (*Avena sativa*), hay, highland barley, and concentrate as energy diet. The intention of supplementing concentrate in the feed was to increase dietary energy supply, organic matter (OM) digestibility, and BW gain. In the current study, ADG data indicated that this goal was achieved and days of experiment had great influence on ADG over the entire cold season. Similar results had been reported in Holstein steers, in which a significant increase in ADG was observed with an increase in dietary energy level [24]. However, the proportionate increase in ADG from d 30 to d 60 with extra energy concentrate was rather inefficient, suggesting that yaks were approaching their genetic growth limit. Kertz et al. [25] reported that ADG in the range from 0.82 to 0.93 kg/d could linearly increase BW and improve productive efficiency of dairy heifers after weaning. Our published data indicated that increasing dietary energy level expedited the fatty acid synthesis and resulted in intramuscular fat deposition through upregulation of lipogenic genes expression in Gannan yak [8], which was beneficial to improve the growth performance. The ADG in the HE group of our current study, however, was slightly lower than in the ME group from day 0 to day 60 because excessive energy intake was not deposited completely in yaks as we expected, indicating that ADG will not increase with the increase in dietary energy level after an appropriate energy level is achieved.

The growth of animal is a complex process, in which fierce competition between anabolism and catabolism exists including a series of physiological and biochemical activities such as glucose and amino acid utilization, intra- and extracellular synthesis or dissociation of protein and fat, all of these reactions are regulated by many factors [26]. In the current study, the average energy intake for yak in LE, ME, and HE dietary groups were adjusted to 11.66 MJ/d, 12.15 MJ/d, and 12.83 MJ/d, respectively. The obtained data of ADG during entire experimental period for each group was lower than 1.0 kg/d. These results indicated that feed energy conversion rate in yak was lower than the recommendatory values because the average temperature of the barn was lower in Qinghai-Tibetan Plateau (data shown in Animal care and experimental site section) and more energy should be used to maintain normal metabolic activities during the experiment. Previous study demonstrated that high dietary energy influenced normal rumen fermentation and decreased fiber degradation ability, resulted in a lot of forage accumulation in the rumen and reduced feed intake [27]. Schwartzkopfgenswein et al. [28] reported that ruminal pH less than 5.5 resulted in decrease of cellulolytic bacteria and increase in amylolytic bacteria when more nonstructural carbohydrates were present in cattle. In our current study, corn is the primary energy source in LE, ME and HE diets, which was fermented in yak rumen to generate volatile fatty acids (VFA). With soaring dietary energy level, content of VFA increased and pH value decreased in rumen, in turn suppressing the normal activities of cellulose degrading bacteria so more highland barley hay stagnated in rumen and affected the feed intake of yak. Feed conversion ratio of yaks present in LE group were higher than ME and HE groups, indicating that yaks in ME and HE groups had higher feed utilization efficiency, which is consistent with the previous studies [9,24].

The somatotrophic axis, including GH, IGF-1, GHR, and IGFBP-3, has a multifunctional role in the metabolism and physiology of mammals [29]. Injection of exogenous bovine GH increased ADG in young cow while BW and ADG showed positive correlation with serum GH concentrations [30]. The increase in concentrations of serum GH and IGF-1 in Holstein heifers was observed with an increase in CP:ME ratio [31], while Sejrsen et al. [32] reported that provision of higher dietary nutrition level feedstuff to heifers decreased the serum concentration of GH. An increase in blood GH concentrations and decrease in IGF-1 concentrations was subjected to feed restrictions [33,34] and opposite response i.e., decrease in serum GH level and increase in IGF-1 level was recorded in beef heifers [35] and crossbred cows [36] when feed was offered to them. In the present study, GH concentration in yak on day 30 and day 60 were higher than those fed medium and high-energy diets. Dietary energy level did not significantly affect serum IGF-1 concentration in yak on day 30, however its concentration was higher in ME group LE and HE groups on day 60. This implies that high energy diet speeds up recovery process in yak body by decreasing the synthesis of protein and increasing fat deposition. It is well known that GH promotes proteosynthesis and lipolysis, so the function of GH in this situation was reduced and its serum concentration was decreased. Purchas et al. [30] demonstrated that serum GH concentration was negatively correlated with BW and ADG, which is similar to our results. It was reported that long term feed restriction increased hepatic GHR expression and induced down-regulation of hepatic GHR mRNA abundance while in muscles up regulation was occurred [37,38]. The hepatic expression of GHR in yak offered with low energy diet was higher as compared to medium and high energy diets, and negative correlation between GHR expression and ADG was observed. It is due to the fact that GH regulates animal growth through binding to GHR and concentration of GH and relative expression of GHR are altered synchronously.

Generally, GH combines with GHR to stimulate target tissues to synthesize IGF-1 and its synthesis in liver is mainly modulated at the transcriptional level and stimulates the hepatic expression of IGFBP-3 [39,40]. It was reported that the serum IGF-1concentration and hepatic expression of IGF-1 and IGFBP-3 had same variation tendency and serum concentration of IGF-1 was increased by the increase in GH concentration [41,42]. We observed that serum concentration of IGF-1elevated with an increase in dietary energy level on day 30 and day 60, which is in agreement with the previous reports in dairy cows [43,44] and heifers [45]. The variation trend of IGF-1 concentration was opposite to GH and higher IGF-1 concentration was observed in the ME group on day 60 and hepatic expression of IGF-1 and IGFBP-3 were increased with an increase in dietary energy level. These results indicated that the axis between GH and IGF-1 was broken by high energy diet and IGF-1 concentration was significantly affected by dietary energy level. Bishop et al. [46] demonstrated that serum IGF-1 concentrations was significantly different between high and low feed conversion ration progeny groups (125.12 vs. 89.52 ng/mL), ADG in high conversion ration progeny group was higher than in low conversion ration progeny group of Angus beef cattle, which is consistence with our results. In addition, feeding forage-concentrate diet had higher serum IGF-1 concentration and positive correlation was found between plasma IGF-1 level and growth rate in young cattle [47]. Similarly, serum IGF-1 level exhibited noteworthy positive correlation with ADG on day 30 and day 60 and positive correlation between relative expression of IGFBP-3 and ADG was also observed, indicating that IGF-1 and IGFBP-3 can improve growth performance of yak when fed with energy diets.

## 5. Conclusions

The current study assessed the effects of serum concentrations of GH, IGF-1, and hepatic expression of GHR, IGF-1, and IGFBP-3 on growth performance of yaks. The results indicated that ADG, feed conversion ratio, serum IGF-1 concentration and relative expression of IGF-1 and IGFBP-3 was increased with an increase in dietary energy level, while serum GH concentration and hepatic GHR expression decreased. Serum IGF-1 concentration, hepatic expressions of IGF-1 and IGFBP-3 showed positive correlation with ADG, but serum GH concentration and GHR expression were negatively correlated with ADG. Yaks offered with medium energy diet (NEg: 6.2 MJ/kg) showed superior feed conversion ratio by gaining 0.883 kg/d and displayed better growth performance during experiment, this study will provide the reference for improvement of supplementary feeding strategy for yak in the cold season.

## Figures and Tables

**Table 1:** Nutrients composition of three energy diets used during experiment.

Item	Treatment ^1^		
	LE (%)	ME (%)	HE (%)
Ingredient, % of DM			
Corn	32.00	44.00	56.00
Corn germ	28.00	20.00	12.00
Wheat bran	4.80	4.80	4.80
DDGS ^2^	9.00	5.00	3.00
Prickly ash seed	4.00	4.00	4.00
Cottonseed meal	12.00	12.00	10.00
Soybean meal	5.30	5.30	5.30
White stone powder	2.00	2.00	2.00
Dicalcium phosphate	0.60	0.60	0.60
Urea	0.50	0.50	0.50
Sodium bicarbonate	1.00	1.00	1.00
Premix ^3^	0.80	0.80	0.80
Nutrient composition, % of DM			
Crude protein	16.53	16.74	17.21
Crude fat	3.73	4.18	5.57
Acid detergent fiber	4.54	4.14	3.72
Neutral detergent fiber	15.93	13.15	12.32
Phosphorus	0.31	0.34	0.36
Calcium	0.64	0.84	0.75
NEg ^4^ (MJ/kg)	5.5	6.2	6.9

^1^ LE, low energy level; ME, medium energy level; HE, high energy level; ^2^ DDGS, distillers dried grains with solubles; ^3^ Premix was provided per kilogram of total diet DM, and the composition is as follow: 22,520 IU of vitamin A, 1,920 IU of vitamin D3, 18 IU of vitamin E, 0.36 IU of vitamin K3, 21.2 mg of D-calcium pantothenate, 9 mg of Cu, 132.8 mg of Zn, 240 mg of Fe and 8 mg of Mn, 0.28 mg of Co; ^4^ NEg is calculated according to Feeding Standard of Beef Cattle (NY/T 815-2004), others were measured values.

**Table 2:** Names, primer sequences (F = forward, R = reverse), accession number, product size of candidate genes.

Gene Name ^1^	Primer Sequence (5′→3′)		Accession	Product Size
β-actin	F	ACCATCGGCAATGAGCG	DQ838049	150bp
	R	CACCGTGTTGGCGTAGAG		
GHR	F	AATGTGGTCCTTTCCC	NM176608	116bp
	R	CAGAAGTAAGCGTTGTCC		
IGF-1	F	ATGCCCATCACATCCTCC	NM001077828	254bp
	R	TACATCTCCAGCCTCCTCA		
IGFBP-3	F	ACAGACACCCAGAACTTCTCCTC	NM174556	236bp
	R	GTTCAGGAACTTGAGGTGGTTC		

^1^ GHR, growth hormone receptor; IGF-1, insulin-like growth factor 1; and IGFBP-3, insulin-like growth factor binding protein 3.

**Table 3:** Growth performance of yaks fed with different dietary energy levels.

Item ^1^	Time ^2^	Treatment ^3^			SEM	*p*-Value			
		LE	ME	HE		Treatment I ^4^	Treatment II ^5^	Time	Treatment × Time
BW, kg	1~60	296.8	305.6	304.3	6.264	0.002		<0.001	0.009
	1	276.0	277.0	275.2	2.555		0.785		
	30	301.0 ^b^	310.2 ^a^	310.8 ^a^	3.427		0.037		
	60	313.4 ^b^	330.0 ^a^	326.8 ^a^	4.357		0.012		
ADG, kg/d	1~60	0.624 ^b^	0.883 ^a^	0.860 ^a^	0.034	<0.001		<0.001	0.021
	1~30	0.833 ^b^	1.107 ^a^	1.186 ^a^	0.041		<0.001		
	30~60	0.413 ^b^	0.660 ^a^	0.533 ^ab^	0.062		0.013		
ADFI, kg/d	1~60	8.116 ^a^	7.958	7.780 ^b^	0.050		0.010		
Feed Conversion Ratio	1~60	13.087 ^a^	9.028 ^b^	9.088 ^b^	0.537		<0.001		

^1^ BW, body weight; ADG, average daily gain; ADFI, average daily feed intake; ^2^ 1~60, mean value of the experiment from day 1 to day 60; 1~30, mean value of the experiment from day 30 to day 60; 30~60, mean value of the experiment from day 30 to day 60; ^3^ LE, low energy; ME, medium energy; HE, high energy; ^4^ *p* value for mixed linear model; ^5^ *p* value for general linear model. Values are presented as mean ± SEM. Within a row, means with different small letter superscripts differ significantly (*p* < 0.05).

**Table 4:** Pearson correlation coefficient between serum hormone levels and growth performance on day 30 and day 60 in yak.

Item ^1^	Time	BW		ADG	
		d 30	d 60	d 30	d 60
GH	d 30	−0.175		−0.493	
	d 60		−0.302		−0.525 *
IGF-1	d 30	0.200		0.606 *	
	d 60		0.338		0.674 **

^1^ BW, body weight; ADG, average dairy gain; GH, growth hormone; IGF-1, insulin-like growth factor 1. Pearson correlation coefficient is used across all treatments. Number of observations = 15. * *p* < 0.05, ** *p* < 0.01.

**Table 5:** Pearson correlation coefficient between relative genes expression and growth performance in yak.

Item ^1^	GHR	IGF-1	IGFBP-3
BW	−0.250	0.474	0.463
ADG	−0.531 *	0.431	0.715 **

^1^ BW, body weight; ADG, average dairy gain; GH, growth hormone; IGF-1, insulin-like growth factor 1. Pearson correlation coefficient is used across all treatments. Number of observations = 15. * *p* < 0.05, ** *p* < 0.01.
